# Evaluation of Apoptosis and Cytotoxicity Induction Using a Recombinant Newcastle Disease Virus Expressing Human IFN-γ in Human Prostate Cancer Cells In Vitro

**DOI:** 10.3390/biomedicines13071710

**Published:** 2025-07-14

**Authors:** Aldo Rojas-Neyra, Katherine Calderón, Brigith Carbajal-Lévano, Gloria Guerrero-Fonseca, Gisela Isasi-Rivas, Ana Chumbe, Ray W. Izquierdo-Lara, Astrid Poma-Acevedo, Freddy Ygnacio, Dora Rios-Matos, Manolo Fernández-Sánchez, Manolo Fernández-Díaz

**Affiliations:** Research and Development Laboratories, FARVET S.A.C., Carretera Panamericana Sur N° 766 Km 198.5, Chincha Alta 11702, Peru; kcalderon@farvet.com (K.C.); acarbajal@farvet.com (B.C.-L.); gloria.guerrero@farvet.com (G.G.-F.); gisasi@farvet.com (G.I.-R.); zatrixa@gmail.com (A.C.); r.izquierdolara@erasmusmc.nl (R.W.I.-L.); astrid.acevedo@farvet.com (A.P.-A.); freddy.a@farvet.com (F.Y.); dora.rios@farvet.com (D.R.-M.); manoloj@farvet.com (M.F.-S.); farvet@farvet.com (M.F.-D.)

**Keywords:** Newcastle disease virus, DU145, oncolytic, apoptosis

## Abstract

**Background/Objectives**: Prostate cancer is the second most common type of cancer diagnosed in men. Various treatments for this cancer, such as radiation therapy, surgery, and systemic therapy, can cause side effects in patients; therefore, there is a need to develop new treatment alternatives. One promising approach is virotherapy, which involves using oncolytic viruses (OVs), such as the recombinant Newcastle disease virus (rNDV). **Methods**: We used the lentogenic rNDV rLS1 strain (the control virus) as our backbone to develop two highly fusogenic rNDVs: rFLCF5nt (the parental virus) and rFLCF5nt-IFN-γ (rFLCF5nt expressing human interferon-gamma (IFN-γ)). We evaluated their oncolytic properties in a prostate cancer cell line (DU145). **Results**: The results showed the expression and stability of the IFN-γ protein, as confirmed using Western blotting after ten passages in specific pathogen-free chicken embryo eggs using the IFN-γ-expressing virus. Additionally, we detected a significantly high oncolytic activity in DU145 cells infected with the parental virus or the IFN-γ-expressing virus using MTS (a cell viability assay) and Annexin V-PE assays compared with the control virus (*p* < 0.0001 for both). **Conclusions**: In conclusion, our data show that IFN-γ-expressing virus can decrease cell viability and induce apoptosis in human prostate cancer in vitro.

## 1. Introduction

Cancer is one of the leading causes of death in the world, causing approximately 10 million deaths in 2020. The most common cancer types are breast, lung, colon, and prostate, according to the World Health Organization (WHO) [1], while prostate cancer is the second most frequent cancer diagnosis in men and the fifth leading cause of death worldwide [2]. It is commonly known that there are many different types of cancer treatments, including surgery, radiation therapy, and/or systemic therapy, which can cause side effects in patients during active treatment, even months or years later. The most common side effects are pain, fatigue, and emotional distress [3]. However, there is also interest in developing new alternatives, one of which is virotherapy through the use of oncolytic viruses (OVs).

Many in vitro, pre-clinical, and clinical trials have shown the oncolytic properties of Newcastle disease virus (NDV) in multiple types of cancer [4,5,6]. NDV is selective toward tumor cells due to defects in the antiviral responses and the activation of apoptotic pathways [7]. In addition, the ubiquitous NDV sialic acid receptor enables the use of NDV against various types of cancer [8,9]. Furthermore, this OV has other advantages: it is not pathogenic to humans, only causes a mild fever for one day, and in some cases, induces limited conjunctivitis [10]. Notably, the majority of the human population is seronegative, avoiding the problem of pre-existing immunity in many candidate OVs and the undetectable genetic recombination rate in the cells infected with NDV [8,9]. Overall, these qualities could make NDV an ideal OV candidate.

NDV causes Newcastle disease (ND) in poultry. Also known as *Avian orthoavulavirus* 1 (AOAV-1), NDV is a member of the genus *Orthoavulavirus* and the family *Paramyxoviridae* [11]. The genome of NDV is a single-stranded, negative-sense ribonucleic acid (RNA), with a size of approximately 15 kilobases (kb), encoding six structural proteins, nucleocapsid protein (NP), phosphoprotein (P), matrix protein (M), fusion protein (F), haemagglutinin–neuraminidase protein (HN), and large protein (L), as polymerase RNA [12]. NDV is classified into five pathotypes according to the clinical signs produced in infected chickens: viscerotropic velogenic, neurotropic velogenic, mesogenic, lentogenic, and asymptomatic [10]. Recombinant NDV (rNDV) has been used as a vaccine vector to express virus antigens from other species to immunize chickens, pigs, cattle, cats, dogs, and humans [13,14].

Strategies exist to enhance the oncolytic effectiveness of rNDV. For instance, other researchers have modified the genetic sequence of the NDV F protein to include a polybasic cleavage site; this modification improved the fusogenic activity of a lentogenic nonpathogenic chicken vaccine strain (Hitchner B1 of NDV) in a mouse colon carcinoma cell line (CT26 cells) [15]. Studies have shown that mice treated with this highly fusogenic NDV exhibit a significant reduction in CT26 tumor growth compared with mice treated with the wild-type virus [15].

As noted, NDV, an OV, can be genetically modified to enhance its oncolytic activity. For example, NDV has been used as an excellent carrier of genes expressing interferons, pro-inflammatory cytokines, and antitumor factors [13]. These modifications involve expressing immunostimulatory heterologous genes, such as interleukin-12 (IL-12) [16], granulocyte/macrophage colony-stimulating factor (GM-CSF) [17], interferon-gamma (IFN-γ), interleukin-2 (IL-2), or tumor necrosis factor-alpha (TNF-α) [15] in various human cancers. IFN-γ is an antitumor immunomodulator produced primarily by T lymphocytes, natural killer (NK) cells, and natural killer T (NKT) cells [18], and it has garnered significant interest from researchers. For instance, a study investigating Meth A tumors in mice revealed that endogenously produced IFN-γ was essential for decreased tumor growth and lipopolysaccharide (LPS)-induced tumor rejection [19]. Furthermore, the antitumor response induced by IFN-γ was demonstrated in an experimental tumor therapy using the cytokine IL-12. This study showed that the protective effects of IL-12 in tumor-bearing mice were diminished by treatment with neutralizing IFN-γ-specific monoclonal antibodies (mAbs) [18]. Additionally, IFN-γ has exhibited anti-proliferative properties in various tumor cell types. This effect is mediated through IFN-γ/Stat1-inducible gene products that inhibit cell cycle progression [20]. For example, IFN-γ can inhibit CDK-2 and CDK-4 kinase activities by inducing cell cycle inhibitors like P21^WAF1^ and P27^Kip1^ in Daudi cells [21]. In addition, the IFN-γ/Stat1 signaling pathway induces genes such as caspase-1 [22] and Fas and Fas ligand (FasL), which promote tumor cell apoptosis [23].

Therefore, researchers have developed an rNDV expressing murine IFN-γ. Unfortunately, this modification does not significantly alter the virus’s oncolytic activity against colon cancer cells (CT26 cells) in immunocompetent BALB/c mice compared with the original virus [15]. However, another study used a vesicular stomatitis virus expressing murine IFN-γ to reduce the growth of 4T1 mammary adenocarcinoma in immunocompetent BALB/c mice compared with the parental virus; this antitumor efficacy, however, was lost in immunocompromised mice [24]. A recent in vitro study showed that an rNDV expressing human IFN-γ significantly reduced the viability of the human respiratory epithelial cancer cell lines, HEp-2 and A549, compared with the parental rNDV [25]. These studies reveal that the action of IFN-γ could depend on the type of cancer or virus used as a vector.

For this study, we used the genome of a lentogenic rNDV (rLS1) as our backbone. We replaced the dibasic motif in the F protein cleavage site with a polybasic motif to enhance the fusogenic activity and oncolytic properties of the recombinant virus (rFLCF5nt) during the infection of the DU145 prostate cancer cell line. However, as noted, immunostimulators may play a key role in oncolytic activity against various tumors. Therefore, we used the rFLCF5nt virus as a backbone to express human IFN-γ (rFLCF5nt-IFN-γ) and assess its oncolytic properties in DU145 cells in vitro.

## 2. Materials and Methods

### 2.1. Cell Lines, Viruses and Animals

A DU145 prostate cancer cell line was purchased from the ATCC (Manassas, VA, USA) and maintained in Dulbecco’s Modified Eagle’s Medium F-12 (1:1) 1× (DMEM 1×) (catalog no. SH30004.04, HyClone, Logan, UT, USA) supplemented with 10% heat-inactivated fetal bovine serum (FBS) (catalog no. SH30066.03, ThermoFisher Scientific, Waltham, MA, USA). African green monkey kidney cells (Vero) and cells derived from chicken fibroblasts (DF-1) were purchased from the ATCC (Manassas, VA, USA) and maintained in DMEM 1× supplemented with 5% FBS. The cell lines were cultivated in the presence of 1× antibiotic-antimycotic solution (catalog no. 15240062, ThermoFisher Scientific, Waltham, MA, USA) at 37 °C under a 5% CO_2_ atmosphere. The cell lines were used to characterize rNDV and evaluate its oncolytic properties.

The rLS1 virus was provided by the research and development laboratories of FARVET S.A.C. (Chincha–Peru). The rFLCF5nt and rFLCF5nt-IFN-γ viruses were developed in this study using the pFLC-LS1 plasmid as a backbone. The viruses were amplified in 9- or 10-day-old specific pathogen-free (SPF) white leghorn chicken embryonated eggs (Charles River Avian Vaccine Services, Norwich, CT, USA). The mean death time (MDT) was also evaluated in 9-day-old SPF chicken embryonated eggs.

### 2.2. Plasmid Construction, Virus Recovery and Pathogenicity

**Figure 1:**
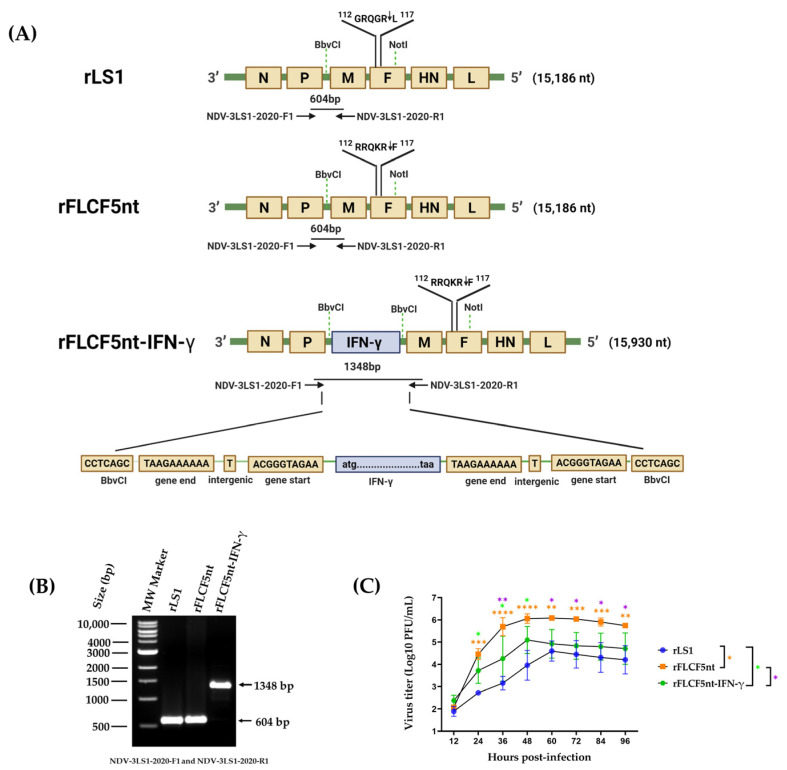
Characterization of recombinant Newcastle disease viruses. (**A**) Schematic representation of the generation of the rFLCF5nt and rFLCF5nt-IFN-γ (IFN-γ-expressing virus) viruses, based on modification of the fusion (F) gene cleavage site and insertion of a human IFN-γ expression cassette. (Illustration created with BioRender.com; not drawn to scale). (**B**) RT-PCR analysis confirming the insertion of the IFN-γ cassette. Amplicon sizes: 604 bp for rLS1 and rFLCF5nt; 1348 bp for rFLCF5nt-IFN-γ. MW: molecular weight marker. (**C**) Viral replication kinetics of rLS1, rFLCF5nt, and rFLCF5nt-IFN-γ in DU145 cells at an MOI of 0.001, measured using plaque assay. Statistical significance: * *p* < 0.05, ** *p* < 0.01, *** *p* < 0.001, **** *p* < 0.0001.

To generate the new recombinant viruses in this study, the previously constructed pFLC-LS1 plasmid [26] was used as the backbone, which contained the plus sense genome sequence (15,186 nucleotides (nt)) of a lentogenic NDV strain (rLS1) with an intracerebral pathogenicity index (ICPI) value of 0.1 and an MDT of 108 h, previously rescued and characterized in another study [27]. In the pFLC-LS1 plasmid, five nucleotides in the cleavage site of the F gene were changed to increase the fusogenic activity, where the dibasic motif ^112^GRQGRL^117^ was replaced by polybasic motif ^112^RRQKRF^117^, by replacing the region between *BbvCI* and *NotI* restriction sites with a chemically synthesized fragment. The resulting plasmid was named pFLCF5nt (19,319 nt), containing the plus sense genome sequence of NDV (15,186 nt), which generated the rFLCF5nt virus (Figure 1A).

The expression cassette of the IFN-γ protein was chemically synthesized using GenScript (Piscataway, NJ, USA), which contains, from 5′ to 3′, the gene start (GS) transcriptional signal of the M gene of pFLC-LS1 plasmid, the human IFN-γ gene (GenBank accession no. NM_000619.3), and the gene end (GE) transcriptional signal of the P gene of pFLC-LS1 plasmid. This expression cassette was flanked by *BbvCI* restriction sites to be inserted into the pFLCF5nt plasmid through the *BbvCI* site in the non-coding region between the P and M genes. The resulting plasmid was named pFLCF5nt-IFN-γ (20,063 nt), containing the plus sense genome sequence of NDV that carries an IFN-γ gene with a length of 15,930 nt, maintaining the rule of six, which generated the rFLCF5nt-IFN-γ virus (Figure 1A).

The recombinant viruses were recovered using co-transfecting cells as previously described [26]. Briefly, Vero cells were co-transfected with the previously generated supporting plasmids (pCI-N, pCI-P, and pCI-L) [26], and pFLCF5nt and pFLCF5nt-IFN-γ plasmids were used to generate the rFLCF5nt and rFLCF5nt-IFN-γ viruses, respectively. To amplify the viruses, the cell supernatant was inoculated into the allantoic cavity of 9-day-old SPF chicken embryonated eggs and incubated for 4 days. Then, the allantoic fluid (AF) was harvested, clarified, aliquoted, and stored at −80 °C. The genetic maps of the rFLCF5nt and rFLCF5nt-IFN-γ viruses are shown in Figure 1. The presence and replication of the recovery viruses were confirmed with a hemagglutination assay (HA) using 1% chicken red blood cells and a plaque assay. The identity of the viruses was confirmed through reverse transcription polymerase chain reaction (RT-PCR). The pathogenicity of the recovered viruses was evaluated via MDT using 9-day-old SPF chicken embryonated eggs (Charles River Avian Vaccine Services, Norwich, CT, USA) following the standard procedures [28]

### 2.3. Detection via RT-PCR

To verify the genetic sequence of the IFN-γ expression cassette in the rFLCF5nt-IFN-γ genome, viral RNA was extracted from the AF stocks of the rLS1, rFLCF5nt, and rFLCF5nt-IFN-γ viruses using the QIAamp MinElute Virus Spin kit, according to the manufacturer’s instructions, with some modifications. The RT-PCR process was performed on the 2720 Thermal Cycler (Applied Biosystems, Waltham, MA, USA). Complementary deoxyribonucleic acid (cDNA) was generated from RNA using the ProtoScript^®^ II cDNA Synthesis kit (catalog no. M0368, New England Biolabs, Ipswich, MA, USA), according to the manufacturer’s instructions. The cDNA was amplified using high-fidelity deoxyribonucleic acid (DNA) polymerase Master Mix Q5 (catalog no. M0492S, New England Biolabs, Ipswich, MA, USA) with the following specific primers: NDV-3LS1-2020-F1 (5′-GAT CAT GTC ACG CCC AAT GC-3′) and NDV-3LS1-2020-R1 (5′-GCA TCG CAG CGG AAA GTA AC-3′). These bind to the P and M genes, respectively. The thermal cycling protocol comprised an initial denaturation step at 98 °C for 30 s, followed by 35 cycles of 98 °C for 10 s, 65 °C for 15 s, and 72 °C for 1 min 20 s, with a final extension step at 72 °C for 2 min. The amplified genetic sequences were visualized on a 1% agarose gel using a CCD camera Azure c600 imaging system (Azure Biosystems, Inc., Dublin, OH, USA).

### 2.4. In Vitro Viral Replication Properties and Plaque Assay

We compared the infectivity and growth properties between the rLS1, rFLCF5nt, and rFLCF5nt-IFN-γ viruses. DU145 cells were seeded at 90% confluence in 12-well plates and infected with rLS1, rFLCF5nt, or rFLCF5nt-IFN-γ virus at a multiplicity of infection (MOI) of 0.001. Cells were maintained with DMEM containing 2% FBS and incubated at 37 °C with 5%CO_2_. Supernatants of the infected cells were collected at 12, 24, 36, 48, 60, 72, and 96 h post-infection (hpi) and kept at −80 °C. The titers of each collected supernatant were determined using a plaque assay in a DF-1 chicken fibroblast cell line, as described previously [26], with some modifications, where the overlay medium was composed of 1% agarose, 2% FBS, 30 mM MgCl_2_, and 5% AF as a source of exogenous protease. The cells were fixed with formaldehyde and stained with crystal violet. Finally, the plaque-forming units (PFUs) were counted using an Axio Observer A1 fluorescence microscope (Carl Zeiss, Jena, Germany). The viral titers were reported as PFU per milliliter (PFU/mL). The experiment was conducted in triplicate.

### 2.5. Immunofluorescence Assay (IFA)

To examine the IFN-γ protein expression, DF-1 cells were seeded into a 24-well plate at a density of 0.16 × 10^6^ cells per well and incubated for 6 h. Then, the cells were infected with the rFLCF5nt or rFLCF5nt-IFN-γ virus at an MOI of 0.00001. Cells were maintained with DMEM containing 2% FBS and incubated at 37 °C with 5% CO_2_. At 48 hpi, the cells were fixed with 4% paraformaldehyde for 30 min at room temperature (RT). Then, the monolayer was washed three times with Dulbecco’s phosphate-buffered saline 1× (DPBS 1×) and permeabilized with 0.3% TRITON^®^ X-100 (catalog no. 648463, Merck, Darmstadt, Germany) for 15 min at RT. Then, the monolayer was incubated with 5% Bovine Serum Albumin (BSA) (catalog no. A7030-500G, Sigma-Aldrich, St. Louis, MO, USA) in DPBS 1× for 3 h at RT. To detect IFN-γ protein expression, the monolayer was incubated with a rabbit polyclonal antibody anti-interferon gamma protein (1:200) (catalog no. ab9657, Abcam, Cambridge, MA, USA) for 1 h 30 min at RT, followed by donkey antibody anti-rabbit IgG H&L conjugated to Alexa Fluor^®^ 594 (1:250) (catalog no. ab150072, Abcam, Cambridge, MA, USA) for 60 min at RT. Afterward, to detect the expression of NDV proteins, the monolayer was incubated with an anti-NDV chicken serum (1:200) (#10100486, Charles River Avian Vaccine Services, Norwich, CT, USA) for 1 h 30 min at RT, followed by goat antibody anti-chicken IgY H&L conjugated to Alexa Fluor^®^ 488 (1:1000) (catalog no. ab150169, Abcam, Cambridge, MA, USA) for 60 min at RT. Finally, the cell nuclei were stained with 4′,6-diamidino-2-phenylindole (DAPI) (catalog no. ab104139, Abcam, Cambridge, MA, USA) for 5 min and observed under an Axio Observer A1 fluorescence microscope (Carl Zeiss, Jena, Germany). Digital images were taken at 400× magnification (Scale bar = 100 µm) with an AxioCam MRc5 camera (Carl Zeiss, Jena, Germany).

### 2.6. Western Blotting

To evaluate the IFN-γ protein expression, DF-1 cells were seeded into a 6-well plate at a density of 0.5 × 10^6^ cells per well and incubated for 16 h. Then, the cells were infected with the rFLCF5nt or rFLCF5nt-IFN-γ virus at an MOI of 0.0001; in addition, non-infected cells were evaluated in this assay. The cells were maintained with DMEM containing 2% FBS and incubated at 37 °C with 5% CO_2_. At 48 hpi, the cells from the wells were harvested, lysed, and analyzed via Western blot assay using a rabbit polyclonal antibody anti-interferon gamma protein (3/5000) (catalog no. ab9657, Abcam, Cambridge, MA, USA) as a primary antibody and an anti-rabbit IgG conjugated to horseradish peroxidase (HRP) (2/5000) (catalog no. A01827, GenScript Laboratories, Piscataway, NJ, USA) as a secondary antibody. NDV F protein expression in the cell pellet was detected using a rabbit polyclonal antibody against NDV F protein (10/5000) (GenScript Laboratories, Piscataway, NJ, USA) as a primary antibody and an anti-rabbit IgG conjugated to HRP (2/5000) (catalog no. A01827, GenScript Laboratories, Piscataway, NJ, USA) as a secondary antibody. Additionally, as a loading control in cell lysates, the beta-actin protein was detected using a mouse monoclonal antibody against beta-actin (5/5000) (catalog no. ab8226, Abcam, Cambridge, MA, USA) as a primary antibody and a goat anti-mouse IgG conjugated to HRP (3/5000) (catalog no. A00160, GenScript Laboratories, Piscataway, NJ, USA) as a secondary antibody. The protein bands were visualized with a CCD camera Azure c600 imaging system (Azure Biosystems, Inc., Dublin, OH, USA).

### 2.7. Quantitative Measurement of Interferon-Gamma in Cell Culture Supernatant Samples

The concentration of IFN-γ protein in the supernatants of the DU145 cell line infected with IFN-γ-expressing virus (rFLCF5nt-IFN-γ) was detected and quantified using a human interferon-gamma SimpleStep ELISA Kit (catalog no. ab174443, Abcam, Cambridge, MA, USA), according to the manufacturer’s protocol. DU145 cells were seeded in 12-well plates at a density of 0.15 × 10^6^ cells per well. The next day, the cells were infected with rFLCF5nt or rFLCF5nt-IFN-γ at MOIs of 0.1 and 0.01, and uninfected tumoral cells were used as a negative control. Cells were maintained with DMEM containing 2% FBS and incubated at 37 °C with 5% CO_2_. Then, the supernatants were collected at 24, 48, and 72 hpi and centrifuged at 2000× *g* for 10 min at 4 °C to remove debris. A standard curve was generated using a series dilution of lyophilized recombinant human interferon-gamma. Then, 50 µL of standard and samples were added to the appropriate wells of a 96-well plate. Next, 50 µL of antibody cocktail was loaded onto each well, and the plate was covered and incubated for 1 h in the dark at RT on a microplate mixer (catalog no. 8182-2019, USA Scientific, Ocala, FL, USA) at 400 revolutions per minute (rpm). All wells were washed 3 times with 1× wash buffer solution (350 µL/well). Afterward, 100 µL of TMB was added to each well, and the microplate was covered and incubated for 10 min at 400 rpm. Finally, 100 µL of stop solution was added, and the absorbance was read using a spectrophotometer microplate reader (Biotek Instruments Inc., Winooski, VT, USA) at a 450 nm wavelength. The experiment was conducted in duplicate.

### 2.8. Genetic Stability of the rFLCF5nt-IFN-γ Virus

To evaluate genetic stability, the rFLCF5nt-IFN-γ virus was sub-cultivated in 9-day-old SPF chicken embryonated eggs up to passage 10. Then, the viral RNA was extracted from the viruses, and the presence of the insert’s genetic sequence was verified via RT-PCR using the primers NDV-3LS1-2020-F1 and NDV-3LS1-2020-R1 (Figure 1) at passages 1, 5, and 10. Furthermore, IFN-γ protein expression was evaluated using the lysates of DF-1 cells infected with rFLCF5nt-IFN-γ virus at passages 1, 5, and 10 via Western blotting using a specific antibody against IFN-γ protein, as previously described. In the assay, the rFLCF5nt-IFN-γ virus was compared to the rFLCF5nt parental virus.

### 2.9. Fusogenic Activity Assay

DU145 cells were seeded in 12-well plates at a density of 0.15 × 10^6^ cells per well. The next day, the cells were infected with rLS1, rFLCF5nt, or rFLCF5nt-IFN-γ at an MOI of 0.001. Cells were maintained with DMEM containing 2% FBS and incubated at 37 °C with 5% CO_2_. Then, an immunofluorescence assay was performed, as previously described. At 24 hpi, the monolayer was fixed with 4% paraformaldehyde and permeabilized with 0.3% TRITON^®^ X-100. Then, an anti-NDV chicken serum and goat antibody anti-chicken IgY H&L conjugated to Alexa Fluor^®^ 488 were used to detect the expression of NDV proteins. Afterward, the cell nuclei were stained with DAPI and observed under an Axio Observer A1 fluorescence microscope (Carl Zeiss, Jena, Germany). Digital images were taken at 400× magnification (Scale bar = 100 µm) with the AxioCam MRc5 camera (Carl Zeiss, Jena, Germany). Nuclei in 24 fusion areas were counted to determine the average syncytia size. The fusion index was calculated as the ratio of the total nuclei in multinucleated cells to the total nuclei in the field.

To compare the plaque diameter in cells infected with the viruses, DU145 cells were infected with rLS1, rFLCF5nt, or rFLCF5nt-IFN-γ virus at an MOI of 0.001. Cells were maintained with DMEM containing 2% FBS and incubated at 37 °C with 5% CO_2_. At 48 hpi, the supernatants of the infected cells were collected to perform the plaque assay in DF-1 cells, as previously described. The experiment was conducted in triplicate. In each experiment, the diameters of 28 viral plaques were measured for each virus using ImageJ software (version 1.54g).

### 2.10. Cytotoxicity Assays Using MTS

DU145 cells were seeded in 96-well plates at a density of 1 × 10^4^ cells per well and infected with rLS1, rFLCF5nt, or rFLCF5nt-IFN-γ virus at an MOI of 0.01, 0.1, or 1. Cells were maintained with DMEM containing 2% FBS and incubated at 37 °C with 5% CO_2_. At 48 and 72 hpi, the 3-(4,5-dimethylthiazol-2-yl)-5-(3-carboxymethoxyphenyl)-2-(4-sulfophenyl)-2*H*-tetrazolium (MTS) reagent (catalog no. ab197010, Abcam, Cambridge, MA, USA) was added to the cells and incubated at 37 °C with 5% of CO_2_ for 4 h to evaluate cytotoxicity. At the indicated time, treated cells were analyzed in a spectrophotometer microplate reader (Biotek Instruments Inc., Winooski, VT, USA) to measure absorbance at 490 nm. The experiment was conducted in triplicate.

### 2.11. In Vitro Annexin V-PE Analysis

The quantification of cell apoptosis was performed using the Annexin V-PE Apoptosis Staining/Detection Reagent (catalog no. ab14154, Abcam, Cambridge, MA, USA) to detect the externalization of phosphatidylserine (PS) on the external membrane; in addition, SYTOX blue dead cell stain (catalog no. S34857, ThermoFisher, Waltham, MA, USA) was used for nucleic acid staining to indicate cell membrane integrity to quantify the non-viable cells.

DU145 cells were grown in T-75 flasks and infected with rLS1, rFLCF5nt, or rFLCF5nt-IFN-γ at MOIs of 0.01, 0.1, or 1. Cells were maintained with DMEM containing 2% FBS and incubated at 37 °C with 5% CO_2_. At 48 and 72 hpi, the infected cells were trypsinized and washed with DPBS. Then, the cells were suspended in a mixture of Annexin V buffer/Annexin V-PE and incubated for 15 min at RT. Finally, SYTOX was added and incubated for 5 min at RT. Afterward, the apoptotic cells were detected using a BD FACS Canto II flow cytometer (BD Biosciences, San Jose, CA, USA), and the data were analyzed using FlowJo v10.8.1 (BD Life Sciences, Ashland, OR, USA). The results were expressed as the percentage of early and late apoptotic cells. For each sample, 30,000 events were acquired. The flow cytometry plots showed: Quadrant 1 (% of necrotic cells); Quadrant 2 (% of late-apoptotic cells); Quadrant 3 (% of early-apoptotic cells); and Quadrant 4 (% of viable cells). The experiment was conducted in triplicate.

### 2.12. Chromatin Condensation

The cell nuclei were stained with DAPI to evaluate chromatin condensation, a classical hallmark of apoptosis. DU145 cells were grown in a 24-well plate and infected with rLS1, rLS1F5nt, or rLS1F5nt-IFN-γ at an MOI of 0.001. The cells were maintained with DMEM containing 2% FBS and incubated at 37 °C with 5% CO_2_. Then, an immunofluorescence assay was performed, as previously described. At 24 hpi, the monolayer was fixed with 4% paraformaldehyde and permeabilized with 0.3% TRITON^®^ X-100. Non-infected cells were used as a negative control. Then, the cell nuclei were stained through contact with DAPI for 5 min. Finally, an anti-NDV chicken serum and goat antibody anti-chicken IgY H&L conjugated to Alexa Fluor^®^ 488 were used to detect the expression of NDV proteins and observed under an Axio Observer A1 fluorescence microscope (Carl Zeiss, Jena, Germany). Digital images were taken at 400× magnification (scale bar = 100 µm) and processed with an AxioCam MRc5 camera (Carl Zeiss, Jena, Germany).

### 2.13. Statistical Analysis

All data were analyzed using GraphPad Prism v8.0.1 (GraphPad Software, San Diego, CA, USA). Significant differences between viruses in viral replication kinetics, the cytotoxicity MTS assay, and the apoptotic assay were determined using two-way ANOVA with Tukey’s post hoc test. The data for the fusion index, syncytia size, and average plaque diameter between viruses were statistically compared using one-way ANOVA with Tukey’s post hoc test. The differences were considered significant at * *p* < 0.05, ** *p* < 0.01, *** *p* < 0.001, and **** *p* < 0.0001.

## 3. Results

### 3.1. Generation and Characterization of the New Recombinant Viruses

In the pFLC-LS1 plasmid, which contains the genome of a lentogenic rNDV rLS1 strain (control virus), five nucleotides in the cleavage site of the NDV F gene were changed to increase the fusogenic activity—where the dibasic motif ^112^GRQGRL^117^ was replaced with polybasic motif ^112^RRQKRF^117^—to obtain the pFLCF5nt plasmid. Furthermore, the expression cassette of the IFN-γ protein was inserted into the pFLCF5nt plasmid in the non-coding region between the NDV P and M genes to obtain the pFLCF5nt-IFN-γ plasmid.

The co-transfection of the Vero cells with the supporting plasmids (pCI-N, pCI-P, and pCI-L) and pFLCF5nt or pFLCF5nt-IFN-γ plasmid generated the rFLCF5nt (parental virus) or rFLCF5nt-IFN-γ virus (IFN-γ-expressing virus), respectively (Figure 1A). At 72 hpi, visible plaques with cytopathic effects typical of NDV were observed in the transfected cells, showing successful viral recovery for both viruses. The viruses were amplified by inoculating the supernatant of the transfected cells into 9-day-old SPF chicken embryonated eggs. At 72 hpi, the harvested AF showed positive HA titers between 8 and 9 Log_2_, and a viral titer between 7.5 and 8.5 Log_10_ PFU/mL was demonstrated using the plaque assay for both viruses.

Both the rFLCF5nt and rFLCF5nt-IFN-γ viral constructs replicated and reached high viral titers in vitro without requiring exogenous protease supplementation from AF; the MDTs of the 9-day-old SPF embryos were 57.4 and 91.8 h for the parental virus (rFLCF5nt) and IFN-γ-expressing virus (rFLCF5nt-IFN-γ), respectively. Given these MDT values, the parental virus can be classified as velogenic (MDT value < 60 h), while the IFN-γ-expressing virus can be considered lentogenic (MDT value > 90 h). The results confirmed that the five nucleotides changed in the cleavage site of the F gene generated velogenic characteristics in the rescued rFLCF5nt virus.

### 3.2. Detection Using RT-PCR

The genetic sequence of the IFN-γ expression cassette was inserted into the non-coding region between the P and M genes of the rFLCF5nt-IFN-γ genome, and the presence of the rLS1 and rFLCF5nt viruses was detected via RT-PCR using the following primers: NDV-3LS1-2020-F1 (5′-GAT CAT GTC ACG CCC AAT GC-3′) and NDV-3LS1-2020-R1 (5′-GCA TCG CAG CGG AAA GTA AC-3′). The results showed a fragment of 1348 base pairs (bp) in the rFLCF5nt-IFN-γ genome, verifying the correct insertion into the NDV genome. Furthermore, a 604 bp band was detected in the rLS1 and rFLCF5nt genomes (Figure 1B) (Supplementary Materials, Figure S1).

### 3.3. Viral Replication Properties

The replication properties between the rLS1, rFLCF5nt, and rFLCF5nt-IFN-γ viral constructs were compared in vitro. For this, DU145 cells were seeded into 12-well plates and infected with the viruses at an MOI of 0.001. The supernatants of the infected cells were collected at 12, 24, 36, 48, 60, 72, and 96 hpi and titrated using a plaque assay. rFLCF5nt had significantly higher viral replication than rLS1 control virus at 24 (*p* = 0.0002), 36 (*p* < 0.0001), 48 (*p* < 0.0001), 60 (*p* = 0.0015), 72 (*p* = 0.0007), 84 (*p* = 0.0007), and 96 hpi (*p* = 0.0011). Similarly, the IFN-γ-expressing virus (rFLCF5nt-IFN-γ) exhibited significantly higher viral growth than rLS1 at 24 (*p* = 0.0424), 36 (*p* = 0.0226), and 48 hpi (*p* = 0.0182). Conversely, rFLCF5nt showed significantly higher replication than rFLCF5nt-IFN-γ at 36 (*p* = 0.0023), 60 (*p* = 0.0153), 72 (*p* = 0.0116), 84 (*p* = 0.0215), and 96 hpi (*p* = 0.0346) (Figure 1C).

### 3.4. Expression of the IFN-γ Protein in DF-1 and DU145 Cells Infected with rFLCF5nt-IFN-γ

**Figure 2:**
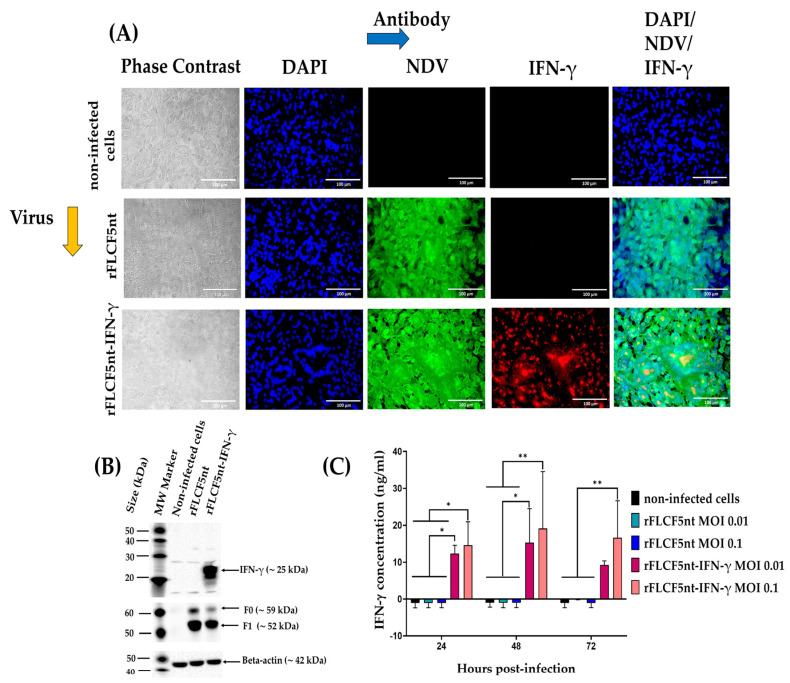
Detection of IFN-γ expression in cells infected with recombinant viruses. (**A**) Immunofluorescence images of DF-1 cells infected with the parental virus (rFLCF5nt) or the IFN-γ-expressing virus (rFLCF5nt-IFN-γ). IFN-γ (red) and NDV proteins (green) were visualized by specific antibody staining; nuclei were counterstained with DAPI (blue). Scale bar: 100 μm. (**B**) Western blot analysis of DF-1 cell lysates showing IFN-γ expression (~25 kDa), NDV fusion proteins (F0 and F1), and beta-actin as loading control. (**C**) Quantification of IFN-γ secretion in DU145 cell culture supernatants by ELISA at 24, 48, and 72 h post-infection. Data represent the median ± SD of two independent experiments. Statistical significance: * *p* < 0.05, ** *p* < 0.01.

IFN-γ protein expression in DF-1 cells infected with the IFN-γ-expressing virus (rFLCF5nt-IFN-γ) was detected via IFA using a rabbit polyclonal antibody anti-interferon gamma protein. NDV protein expression was detected in cells infected with the rFLCF5nt parental virus or rFLCF5nt-IFN-γ virus (Figure 2A).

In lysates of DF1 cells infected with rFLCF5nt-IFN-γ, the IFN-γ protein was detected with a molecular mass of ~25 kDa via Western blotting using a rabbit polyclonal antibody anti-interferon gamma protein. The expression of the NDV F protein was detected in the pellets of cells infected with the rFLCF5nt or rFLCF5nt-IFN-γ virus using a rabbit polyclonal antibody against NDV F protein, where inactive precursor (F0) and the cleaved subunit (F1) of the F protein showed bands of ~59 and ~52 kDa, respectively. The beta-actin protein of ~42 kDa was detected in the lysates of cells infected with the parental virus or IFN-γ-expressing virus and non-infected cells (Figure 2B) (Supplementary Materials, Figure S2).

The concentration of the IFN-γ protein was quantified in the supernatants of DU145 cells infected with rFLCF5nt or rFLCF5nt-IFN-γ virus at MOIs of 0.01 and 0.1 using a human interferon-gamma SimpleStep ELISA Kit, according to the manufacturer’s instructions. The cells infected with the rFLCF5nt-IFN-γ virus at an MOI of 0.01 showed significantly high concentrations of IFN-γ protein at 24 (12.35 ± 2.29 ng/mL), 48 (15.29 ± 9.20 ng/mL), and 72 hpi (9.25 ± 1.15 ng/mL) compared with cells infected with rFLCF5nt and non-infected cells. At an MOI of 0.1, the cells infected with the rFLCF5nt-IFN-γ virus also generated significantly high concentrations of IFN-γ protein at 24 (14.66 ± 6.24 ng/mL), 48 (19.18 ± 15.41 ng/mL), and 72 hpi (16.63 ± 10.01 ng/mL). However, the IFN-γ protein was not detected in the supernatants of cells infected with rFLCF5nt and non-infected cells at 24, 48, and 72 hpi (Figure 2C).

### 3.5. Genetic Stability

**Figure 3:**
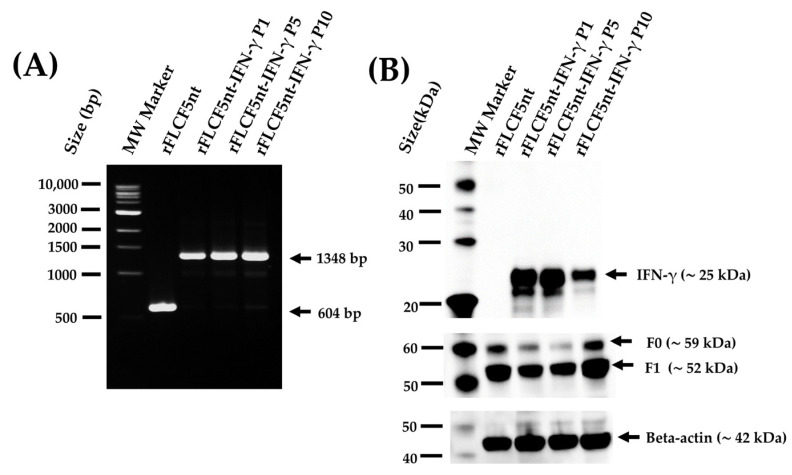
Genetic stability of the IFN-γ-expressing recombinant virus (rFLCF5nt-IFN-γ). (**A**) RT-PCR analysis showing maintenance of the IFN-γ expression cassette across virus passages 1, 5, and 10 in embryonated chicken eggs. The expected amplicon size (1348 bp) is indicated. MW: molecular weight marker. (**B**) Western blot analysis confirming stable IFN-γ protein expression (~25 kDa) in DF-1 cells infected with rFLCF5nt-IFN-γ virus from passages 1, 5, and 10. NDV fusion proteins (F0 and F1) and beta-actin were detected as controls.

The recombinant virus was grown in 9-day-old SPF chicken embryonated eggs up to the tenth passage. RT-PCR using the primers NDV-3LS1-2020-F1 and NDV-3LS1-2020-R1 (Figure 1) showed a 1348 bp band in the rFLCF5nt-IFN-γ genome at the 1st, 5th, and 10th passages. In contrast, the parental virus (rFLCF5nt) displayed a 604 bp band (Figure 3A) (Supplementary Materials, Figure S3). Western blot analysis confirmed IFN-γ protein expression in DF-1 cells infected with the rFLCF5nt-IFN-γ virus from the 1st, 5th, and 10th passages using a specific antibody against the IFN-γ protein (Figure 3B) (Supplementary Materials, Figure S3). Cells infected with the rFLCF5nt virus did not show any IFN-γ protein expression. These results indicate that the viral replication of the rFLCF5nt-IFN-γ did not affect the genetic sequence of the IFN-γ expression cassette in the viral genome up to the 10th passage.

### 3.6. Fusogenic Activity

**Figure 4:**
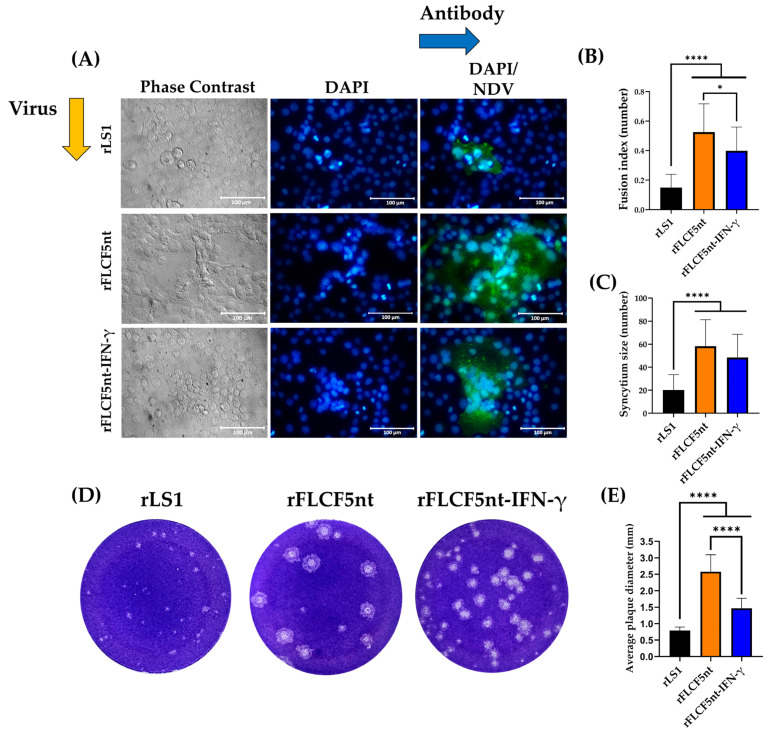
Evaluation of fusogenic activity induced by recombinant viruses. (**A**) Representative immunofluorescence images showing syncytium formation in DU145 cells infected with the parental virus (rFLCF5nt), the IFN-γ-expressing virus (rFLCF5nt-IFN-γ), or the control virus (rLS1). NDV proteins are stained in green; nuclei are counterstained with DAPI (blue). Scale bar: 100 μm. (**B**,**C**) Quantification of fusion index and syncytium size based on 24 randomly selected fusion areas. (**D**,**E**) Plaque size comparison in DF-1 cells infected with the indicated viruses. Data represent mean ± SD of three independent experiments. Statistical significance: * *p* < 0.05, **** *p* < 0.0001.

The syncytium size, fusion index, and plaque diameter were analyzed to measure fusogenic activity in cells infected with the viruses. At 24 hpi, the rLS1 control virus exhibited a significantly low fusion index at 0.15 ± 0.09 compared with 0.40 ± 0.16 for rFLCF5nt-IFN-γ and 0.53 ± 0.19 for rFLCF5nt (*p* < 0.0001, for both). In addition, rFLCF5nt-IFN-γ showed a significantly lower fusion index than rFLCF5nt (*p* = 0.0142) (Figure 4A,B). Similarly, DU145 cells infected with the rLS1 virus showed a syncytium size of 20.17 ± 2.72, which was significantly smaller than the 48.42 ± 4.12 and 58.20 ± 4.71 found in cells infected with rFLCF5nt-IFN-γ and rFLCF5nt (*p* < 0.0001, for both), respectively (Figure 4A,C). On the other hand, the plaque assay in DF-1 cells showed average plaque diameters of 1.46 ± 0.28 mm for rFLCF5nt-IFN-γ and 2.58 ± 0.42 mm for rFLCF5nt, which were significantly larger (*p* < 0.0001 for both) than 0.79 ± 0.12 mm for rLS1. The rFLCF5nt exhibited a significantly larger plaque diameter than rFLCF5nt-IFN-γ (*p* < 0.0001) (Figure 4D,E).

### 3.7. In Vitro Oncolytic Properties of rLS1, rFLCF5nt, and rFLCF5nt-IFN-γ Viruses in DU145 Cells

**Figure 5:**
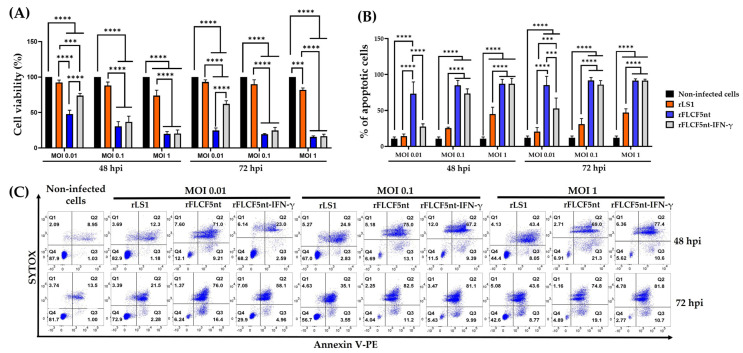
Oncolytic effects of recombinant viruses on DU145 prostate cancer cells. (**A**) Cell viability measurement by MTS assay at 48 and 72 h post-infection (MOIs of 0.01, 0.1, and 1). (**B**) Quantification of apoptotic cells after infection with the parental virus (rFLCF5nt), the IFN-γ-expressing virus (rFLCF5nt-IFN-γ), or control virus (rLS1). (**C**) Representative flow cytometry plots showing apoptosis and necrosis at 48 and 72 h post-infection (hpi). Data represent mean ± SD of triplicate experiments. Statistical significance compared with non-infected cells: *** *p* < 0.001, **** *p* < 0.0001.

Infecting DU145 cells with rLS1 (control virus), rFLCF5nt (parental virus), and rFLCF5nt-IFN-γ (IFN-γ-expressing virus) viruses at various MOIs significantly decreased cell viability. At both 48 and 72 hpi, cells infected with either the parental virus or the IFN-γ-expressing virus at MOIs of 0.01, 0.1, and 1 exhibited a significant reduction in cell viability compared with rLS1-infected cells and non-infected cells (*p* < 0.0001 for both). On the other hand, only at an MOI of 0.01, rFLCF5nt-infected cells showed a significant decrease in cell viability at 48 and 72 hpi compared with rFLCF5nt-IFN-γ-infected cells (*p* < 0.0001). Finally, only at an MOI of 1, the viability of rLS1-infected cells was significantly lower at both 48 and 72 hpi compared with non-infected cells (*p* < 0.0001) (Figure 5A).

In parallel with the reduced cell viability, infection with these viruses also significantly increased apoptosis. At both 48 and 72 hpi, cells infected with rFLCF5nt at an MOI of 0.01, 0.1, and 1 exhibited significantly higher apoptosis than non-infected cells and rLS1-infected cells (*p* < 0.0001 for both comparisons). Similarly, apoptosis was also significantly more prevalent only at MOIs of 0.1 and 1 for rFLCF5nt-IFN-γ-infected cells compared with non-infected cells and rLS1-infected cells (*p* < 0.0001 for both). However, only at 72 hpi, the IFN-γ-expressing virus induced a significantly higher apoptosis percentage at an MOI of 0.01 compared with non-infected cells (*p* < 0.0001) and rLS1-infected cells (*p* = 0.0002). On the other hand, only at an MOI of 1, the rLS1-infected cells showed a significant apoptosis level compared with the non-infected cells (*p* < 0.0001). Furthermore, at an MOI of 0.01, apoptosis was also significantly more prevalent at both 48 (*p* < 0.0001) and 72 hpi (*p* = 0.0002) for rFLCF5nt-infected cells compared with rFLCF5nt-IFN-γ-infected cells (Figure 5B,C).

**Figure 6:**
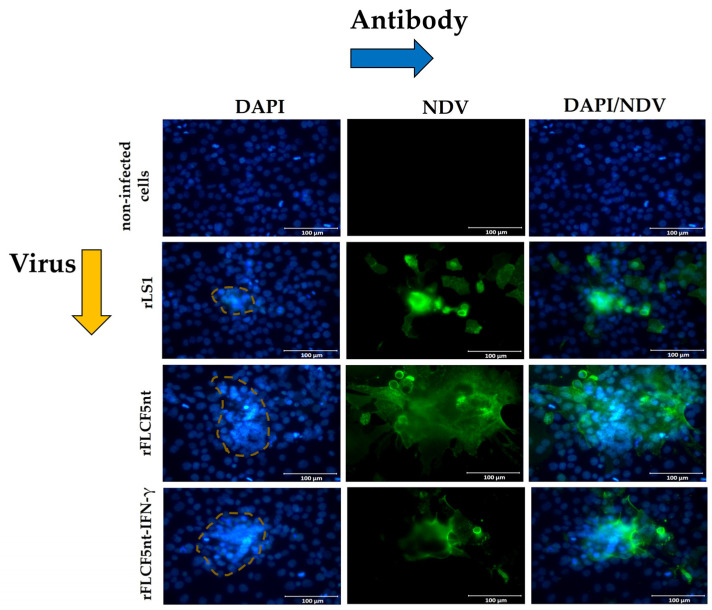
Chromatin condensation induced by recombinant viruses in DU145 cells. Representative fluorescence microscopy images of DU145 cells infected with the parental virus (rFLCF5nt), the IFN-γ-expressing virus (rFLCF5nt-IFN-γ), or the control virus (rLS1). Nuclei were stained with DAPI (blue). Chromatin condensation, indicative of apoptosis, is highlighted with dotted circles. Scale bar: 100 μm.

To assess the impact of viral infection on cell nuclei, we stained them with DAPI. DU145 cells infected with rLS1, rFLCF5nt, or rFLCF5nt-IFN-γ exhibited chromatin condensation at 24 hpi, indicating apoptosis, a hallmark feature. However, rFLCF5nt and rFLCF5nt-IFN-γ showed higher chromatin condensation than rLS1. Conversely, this effect was not observed in non-infected cells (Figure 6).

## 4. Discussion

It has been established in recent decades that various lentogenic, mesogenic, and velogenic strains of NDV possess oncolytic properties. Along with their derived genetic constructs, these strains exhibit potent antitumor activity against a range of human tumor types, such as prostate, lung, colorectal, and breast cancers [4,5]. This is achieved through infection and replication within cancer cells, coupled with the modulation and activation of the host immune response. Cancer cells can have defects at any point along the apoptosis pathway [29], but NDV can cause oncolysis by activating this pathway in these cells [30]. Based on this background, our study evaluated the antitumoral activity of three rNDV viruses—rLS1, rFLCF5nt, and rFLCF5nt-IFN-γ—in a DU145 prostate cancer cell line.

In a past study, a lentogenic rNDV strain (rLS1) containing the dibasic motif ^112^GRQGRL^117^ in the cleavage site of the F gene was generated using reverse genetic technology [26]. For optimal viral replication in vitro, this strain needed allantoic fluid as a source of exogenous protease; furthermore, the ICPI value was 0.1, and the MDT value was 108 h, corresponding to a lentogenic strain [27]. In our study, we used the rLS1 strain (control virus) genome as the backbone. We replaced the dibasic motif ^112^GRQGRL^117^ with the polybasic motif ^112^RRQKRF^117^ in the cleavage site of the rNDV F gene. This modification was intended to enhance the fusogenic activity and oncolytic properties of the virus in DU145 prostate cancer cells. The newly created strain resulting from this modification was named rFLCF5nt (parental virus). Then, the expression cassette of the IFN-γ protein was inserted into the rFLCF5nt genome in the intergenic sequence between the P and M genes of NDV to generate an IFN-γ-expressing virus (rFLCF5nt-IFN-γ). Thus, the rFLCF5nt and rFLCF5nt-IFN-γ viruses did not need exogenous proteases for their optimal viral replication in DU145 cells (in vitro assays). Furthermore, the MDT values were 57.4 and 91.8 h for rFLCF5nt and rFLCF5nt-IFN-γ, respectively; these values indicate a velogenic strain (MDT value < 60 h) and a lentogenic strain (MDT value > 90 h), respectively. Finally, the results of the syncytium size, fusion index, and plaque diameter confirmed that the five nucleotide changes in the cleavage site of the F gene improved the fusogenic activity in both rescued viruses. This strategy has been adopted by other researchers to enhance the fusogenic activity of a nonpathogenic lentogenic chicken vaccine (strain Hitchner B1 of NDV) in a mouse colon carcinoma cell line (CT26 cells) [15]. They achieved this enhancement by genetically modifying the gene that encodes the F protein to contain a polybasic cleavage site.

We evaluated the rFLCF5nt and rFLCF5nt-IFN-γ viruses to show their oncolytic potential in DU145 prostate cancer cells in vitro. The MTS assays revealed that both viruses significantly decreased the viability of DU145 cells. Although the molecular mechanisms of apoptosis in NDV infection are poorly understood across various cancer cell types, apoptotic pathway activation was confirmed in the infected DU145 cells. This was evidenced by the detection of phosphatidylserine externalization on the cell membrane and nuclear condensation. Similarly, other studies have suggested that apoptosis might be the cause of death in oral cancer cells infected with NDV. This conclusion was reached after detecting Annexin V-positive cells, nuclear condensation, and fragmentation [31]. In addition, in our study, the rNDVs expressing highly fusogenic F protein (rFLCF5nt and rFLCF5nt-IFN-γ) showed enhanced oncolytic activity in DU145 prostate cancer cells compared to the control virus (rLS1). Likewise, in another study, a highly fusogenic NDV demonstrated higher antitumor properties in CT26 cancer cells compared with the wild-type virus [15].

Several studies have shown that IFN-γ plays a key role in activating the immune response against the proliferation of various human cancers. For example, IFN-γ production was essential in diminishing Meth A tumor growth in a study in mice [19]. Other investigators have confirmed that the efficacy of IL-12 in mouse tumor therapy depends on the presence of IFN-γ [18]. Here, we constructed a recombinant Newcastle disease virus that expresses human interferon-gamma (rFLCF5nt-IFN-γ) to evaluate its antitumor activity. However, our cell viability assay and apoptotic cell quantification results for the prostate cancer cells did not demonstrate a significant increase in the antitumor activity of the IFN-γ-expressing virus (rFLCF5nt-IFN-γ) compared with the parental virus (rFLCF5nt). This could be because the optimal antitumor activity of IFN-γ depends on the cancer cell type or the presence of specific components of the tumor microenvironment, such as certain immune cells. Therefore, this experimental model may not have been suitable for demonstrating the potential of IFN-γ. The larger genome size of the IFN-γ-expressing virus or the antiviral activity of IFN-γ could be another reason. Maybe the IFN-γ-expressing virus could demonstrate better oncolytic properties in a suitable environment, as shown by in vitro and in vivo assays. Similarly, another study developed an rNDV that expresses murine IFN-γ, but the expression of this immunomodulator did not show a significant change in the oncolytic activity against colon cancer cells (CT26 cells) in immunocompetent BALB/c mice compared with the parental virus [15]. However, another study used a vesicular stomatitis virus to express murine IFN-γ, decreasing the growth of 4T1 mammary adenocarcinoma in immunocompetent BALB/c mice [24]. In a recent in vitro study, an rNDV that expresses human IFN-γ significantly diminished viability in human respiratory epithelial cancer cell lines, HEp-2 and A549, compared with the parental rNDV [25].

The human IFN-γ can promote apoptosis of tumor cells and stimulate immune activation [18]. Therefore, other researchers have developed IFN-γ-expressing oncolytic viruses to improve the antitumoral activity [24,25]. However, IFN-γ may also trigger antiviral responses in infected cells, potentially reducing viral replication and spread, and thus compromising the overall oncolytic effect [32,33]. To date, to our knowledge, there is only evidence that chicken IFN-γ can induce an antiviral environment against NDV in chicken embryo fibroblasts [34]. In our study, the rFLCF5nt-IFN-γ showed lower replicative capacity, fusogenic activity, and oncolytic capacity than the rFLCF5nt parental virus in DU145 cells, which could suggest potential antiviral effects of IFN-γ. Nonetheless, additional investigations are required to demonstrate the antiviral mechanism of human INF-γ against NDV in prostate cancer cells.

In this study, the IFN-γ gene was inserted into the intergenic region between the P and M genes of the rNDV genome using reverse genetic technology, demonstrating once again that the rNDV genome is an excellent vector for expressing immunomodulatory heterologous genes. Similarly, another study used the rNDV genome as a backbone to efficiently express other immunomodulators, including GM-CSF, IFN-γ, IL-2, and TNF-α [15]. rNDV is an expression vector of immunomodulatory genes, but it can also express other genes, such as the severe acute respiratory syndrome coronavirus 2 (SARS-CoV-2) spike protein [35]. On the other hand, we demonstrated intracellular IFN-γ expression in rFLCF5nt-IFN-γ-infected prostate cancer cells at 48 hpi. However, it was also important to determine extracellular IFN-γ expression levels over time for our study design (to measure fusogenic activity, viability, and apoptosis). Therefore, we quantified the concentration of IFN-γ in the supernatants of infected cells at 24, 48, and 72 hpi.

We demonstrated that IFN-γ gene insertion is stable in the rFLCF5nt-IFN-γ genome, as IFN-γ protein expression was detected after 10 passages of the virus in embryonated chicken eggs. Likewise, other investigators have used the rNDV as a vector to carry other foreign genes; for example, Z. Mohamed Amin et al. inserted the IL-12 gene into the rNDV genome and confirmed the expression of IL-12 after 10 passages in SPF embryonated chicken eggs [16].

Between the limitations of our study, we did not demonstrate the functionality of the recombinant IFN-γ protein from rFLCF5nt-IFN-γ-infected cells. Future studies are needed to demonstrate that this IFN-y is functional and can activate immune cells. Additional investigations using other prostate cancer cell lines will also be significant for evaluating the homogeneity of the oncolytic activity of the viruses in this study. Studies involving animal models are required to validate the antitumor potential of these rNDVs.

## 5. Conclusions

This study highlights the development of an rNDV demonstrating the ability to express IFN-γ with high fusogenic activity and oncolytic potential against DU145 cells in vitro. In conclusion, we identified a promising candidate among oncolytic viruses for the treatment of human prostate cancer. However, additional research is required to validate the antitumor efficacy of this rNDV variant in immunocompetent mouse models.

## Data Availability

Data is contained within the article and Supplementary Materials. Further inquiries can be directed to the corresponding author.

## Supplementary Materials

The following supporting information can be downloaded at https://www.mdpi.com/article/10.3390/biomedicines13071710/s1, Figure S1: Characterization of recombinant Newcastle disease viruses; Figure S2: Detection of IFN-γ expression in cells infected with recombinant viruses; Figure S3: Genetic stability of the IFN-γ-expressing recombinant virus (rFLCF5nt-IFN-γ).
