# Supplementary material for: Evaluation of Apoptosis and Cytotoxicity Induction Using a Recombinant Newcastle Disease Virus Expressing Human IFN-γ in Human Prostate Cancer Cells In Vitro
Source: Biomedicines. 2025 Jul 14;13(7):1710. doi: 10.3390/biomedicines13071710 (PMC12292100; doi:10.3390/biomedicines13071710)
Supplement: Supplementary file 1 [file biomedicines-13-01710-s001.zip › biomedicines-3616498-supplementary.pdf]

biomedicines

MDPI

Supplementary Materials

Evaluation of Apoptosis and Cytotoxicity Induction Using a Recombinant Newcastle Disease Virus Expressing Human IFN-γ in Human Prostate Cancer Cells In Vitro

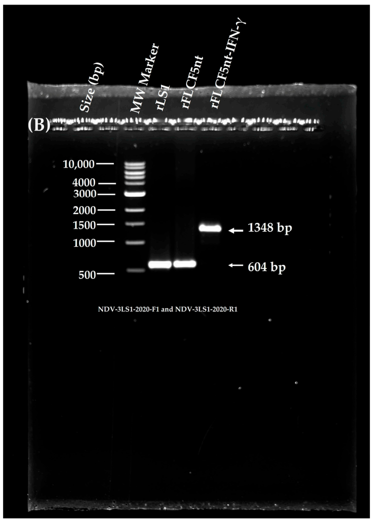

Figure S1. Characterization of recombinant Newcastle disease viruses. RT-PCR analysis confirming the insertion of the IFN-γ cassette. Amplicon sizes: 604 bp for rLS1 and rFLCF5nt; 1348 bp for rFLCF5nt-IFN-γ. MW: molecular weight marker.

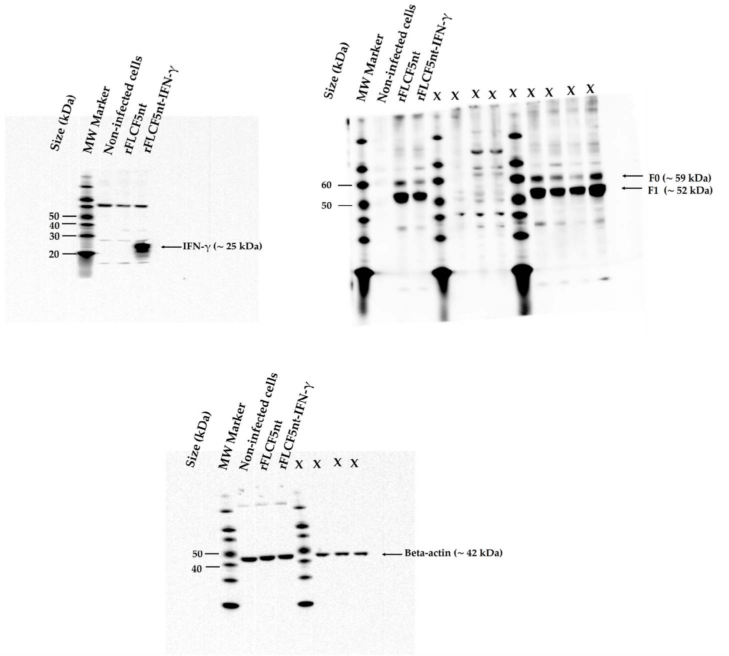

Figure S2. Detection of IFN-γ expression in cells infected with recombinant viruses. Western blot analysis of DF-1 cell lysates showing IFN-γ expression (~25 kDa), NDV fusion proteins (F0 and F1), and beta-actin as loading control.

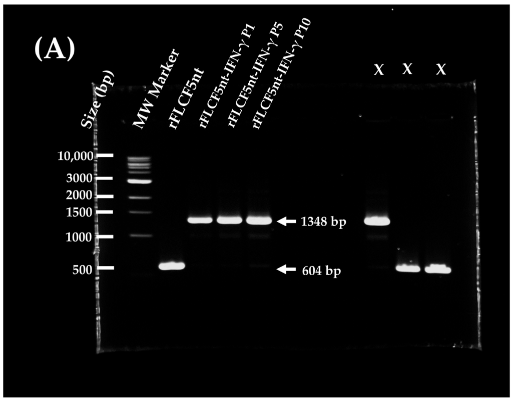

(B)

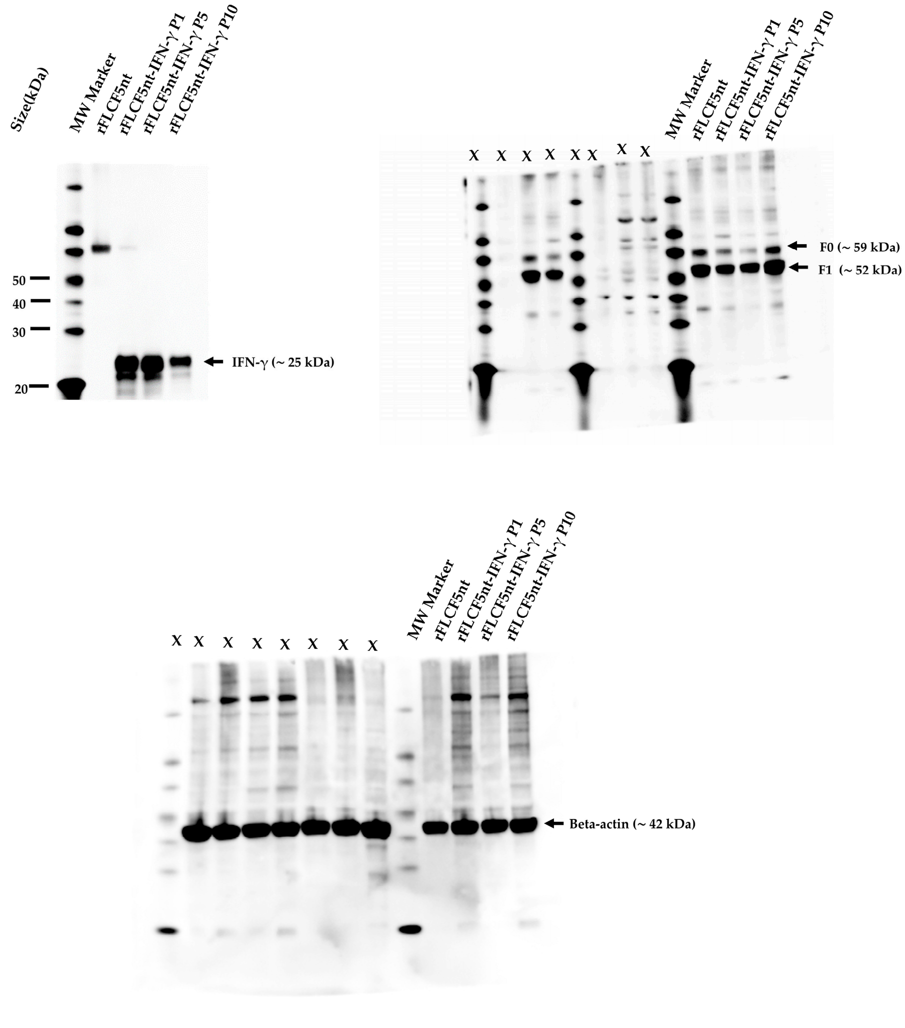

Figure S3. Genetic stability of the IFN-γ-expressing recombinant virus (rFLCF5nt-IFN-γ). (**A**) RT-PCR analysis showing maintenance of the IFN-γ expression cassette across virus passages 1, 5, and 10 in embryonated chicken eggs. The expected amplicon size (1348 bp) is indicated. MW: molecular weight marker. (**B**) Western blot analysis confirming stable IFN-γ protein expression (~25 kDa) in DF-1 cells infected with rFLCF5nt-IFN-γ virus from passages 1, 5, and 10. NDV fusion proteins (F0 and F1) and beta-actin were detected as controls.
